# Unraveling the importance of fabrication parameters of copper oxide-based resistive switching memory devices by machine learning techniques

**DOI:** 10.1038/s41598-023-32173-8

**Published:** 2023-03-25

**Authors:** Suvarna M. Patil, Somnath S. Kundale, Santosh S. Sutar, Pramod J. Patil, Aviraj M. Teli, Sonali A. Beknalkar, Rajanish K. Kamat, Jinho Bae, Jae Cheol Shin, Tukaram D. Dongale

**Affiliations:** 1 Institute of Management, Bharati Vidyapeeth Deemed to be University, Sangli, 416 416 India; 2 Computational Electronics and Nanoscience Research Laboratory, School of Nanoscience and Biotechnology, Shivaji University, Kolhapur, 416004 India; 3 Yashwantrao Chavan School of Rural Development, Shivaji University, Kolhapur, 416004 India; 4 Division of Electronics and Electrical Engineering, Dongguk University, Seoul, 04620 South Korea; 5 Department of Electronics, Shivaji University, Kolhapur, 416004 India; 6 Dr. Homi Bhabha State University, 15, Madam Cama Road, Mumbai, 400032 India; 7 Department of Ocean System Engineering, Jeju National University, 102 Jejudaehakro, Jeju, 63243 South Korea

**Keywords:** Electronic devices, Computational methods, Engineering, Nanoscience and technology

## Abstract

In the present study, various statistical and machine learning (ML) techniques were used to understand how device fabrication parameters affect the performance of copper oxide-based resistive switching (RS) devices. In the present case, the data was collected from copper oxide RS devices-based research articles, published between 2008 to 2022. Initially, different patterns present in the data were analyzed by statistical techniques. Then, the classification and regression tree algorithm (CART) and decision tree (DT) ML algorithms were implemented to get the device fabrication guidelines for the continuous and categorical features of copper oxide-based RS devices, respectively. In the next step, the random forest algorithm was found to be suitable for the prediction of continuous-type features as compared to a linear model and artificial neural network (ANN). Moreover, the DT algorithm predicts the performance of categorical-type features very well. The feature importance score was calculated for each continuous and categorical feature by the gradient boosting (GB) algorithm. Finally, the suggested ML guidelines were employed to fabricate the copper oxide-based RS device and demonstrated its non-volatile memory properties. The results of ML algorithms and experimental devices are in good agreement with each other, suggesting the importance of ML techniques for understanding and optimizing memory devices.

## Introduction

The resistive switching (RS) effect is associated with the switching of materials into two or more stable resistance states by the application of electrical stress. While, these resistance states are stable after the removal of electrical stress, and can be switched frequently by applying the same or opposite bias potential^1^. Owing to these facts, the RS devices become a prominent candidate for memory storage devices^1^. Along with non-volatile memory application, the RS effect can be also utilized in neuromorphic computing^2^, sensing^3^, and logic gates^4^. Among many applications, RS devices are frequently reported for memory storage applications. To compete with the conventional flash memory counterpart, the RS devices need to operate at low switching potential (< 1 V), and show high endurance (> 10^9^ cycles), and long retention (> 10 years) properties^1^. Researchers in this field are trying to optimize the various device parameters to get desired RS properties. Given this, industry and academia are trying to optimize the device synthesis and fabrication process, resistive switching materials, switching layer thickness, the thickness of the top and bottom electrodes, etc.

The optimization of experimental conditions plays a crucial role in tuning the RS properties. Traditionally, each research group fabricates different RS devices and tries to improve its non-volatile memory performance. However, such standalone strategies consumed a lot of capital, resources, and time. To tackle such kind of issues, various data-driven discovery techniques such as machine learning (ML), artificial intelligence, big data analytics, etc. being used in various scientific fields such as material science^5^, energy^6^, environment^7^, and medicine^8^. In most cases, the quality of the data plays an important role in optimizing the device's performance. In some cases, homogeneous datasets are used, owing to the easy availability of raw data. In such cases, the data is generated by simulating various models, or by computing the density functional/first principle theories, or utilizing lab datasets. However, such datasets cannot provide detailed insights into the materials or devices, owing to the absence of intrinsic randomness. Therefore, diverse and heterogeneous data can be the best way to identify the patterns and understand the hidden dynamics of the materials or devices^9,10^. The heterogeneous dataset can be created by collecting the raw data manually/automatically from the peer-reviewed literature. In this way, a versatile dataset is built for the data-driven discovery of different materials and devices.

In recent years, copper oxide is widely investigated material for various applications such as supercapacitors, biosensors, environmental remediation, photocatalysis, non-volatile memory, etc.^11–14^. In the case of non-volatile memory application, the copper oxide-based RS devices showed good performance along with lower device fabrication cost and ease of synthesis^15^, similar to contemporary switching materials^16^. However, early career researchers in this field struggled to optimize the operating voltage, endurance, and retention properties of copper oxide-based RS devices. Therefore, ML techniques can provide synthesis and fabrication guidelines, which help researchers to fabricate high-performance copper oxide-based RS devices. Moreover, we are providing a big picture of this particular field with the help of different ML models and feature importance factors. To this end, we provide different fabrication guidelines to realize high-performance copper oxide-based RS devices by using different ML algorithms. For that, we manually collected raw data from different peer-reviewed research papers published between 2008 to 2022. After cleaning the dataset, we analyzed the data by using statistical methods. Device fabrication guidelines were extracted by implementing classification and regression tree (CART), and decision tree (DT) ML algorithms. For the prediction of RS properties (continuous type features), we used linear model (LM), artificial neural network (ANN), and random forest (RF) algorithms. The categorical type features were predicted by a DT algorithm. Moreover, the feature importance of continuous and categorical features was evaluated by the gradient boosting (GB) algorithm. Finally, ML predictions were validated by fabricating copper oxide-based RS devices.

## Method

### Data collection, preprocessing, and cleaning

In the present work, data were collected by searching the papers related to copper oxide-based resistive switching devices. In particular, we searched the keywords copper oxide + resistive switching in the Scopus database. We found more than 65 research articles published between 2008 to 2022. Among those research articles, some of the research papers were unable to provide relevant information to build a model, hence we remove those data points. The remaining 55 research articles were taken for further consideration. In the present work, 935 data points from peer-reviewed literature were collected and used for statistical analysis, ML-based model development, and prediction. We made an effort to comprehend how the device's physical and electrical characteristics interacted. For instance, different stoichiometries are used in the fabrication methods, hence the oxide is not always the same in all circumstances. Therefore, we collected relevant stoichiometries (CuO, CuO_x_, Cu_2_O, and Cu_x_O) related data in the present work. For statistical analysis and ML data was sorted in tabular form with columns type of materials (TYM), synthesis method (SM), top electrode (TE), the thickness of TE (TTE, in nm), bottom electrode (BE), the thickness of BE (TBE, in nm), the thickness of switching layer (TSL, in nm), type of switching: analog, digital, and both (TSAD), type of switching: unipolar, bipolar and both (TSUB), V_SET_ (V), V_RESET_ (V), endurance cycles (EC, in cycles), retention time (RT, in second), memory window (MW), multilevel resistive states (MRS), conduction mechanism (CM), resistive switching mechanism (RSM), name of the paper, DOI of a paper, and year of publication. As per domain knowledge, we filled in some missing values in the table. It is important to note that the electrical measurement conditions and device parameters such as the slope of the applied voltage ramp, the frequency of the input signal, pulse conditions, current compliance, device area, environmental parameters, etc. affect the RS properties of the device. As most of the literature related to this work has not followed the unified reporting methodology and has not reported all electrical measurement conditions and device parameters, therefore, we have not used 'electrical measurement conditions' and 'few other device parameters' as input for ML analysis. Finally, cleaned data points were used for statistical analysis and ML modeling and predictions.

### Computational details

Operating voltage, endurance, retention, and memory window are the important properties of RS devices. Given this, researchers are trying to optimize these properties by tuning device fabrication parameters. In the present study, we studied how device parameters affect RS performance. The V_SET_, V_RESET_, EC, RT, and MW are the continuous output parameters, therefore, we used the CART algorithm to get the fabrication guidelines. While the DT algorithm was implemented for categorical output features such as TSAD, TSUB, MRS, CM, and RM. The input parameters used to construct ML models are TYM, SM, TE, TTE, BE, TBE, and TSL. For performance prediction, we have used RF, ANN, and LM algorithms. All continuous and discreet parameters are used in prediction models. The feature importance of all features was estimated by the GB algorithm. All ML algorithms were scripted in *R Studio* (version 3.6.2). The *OriginPro 8.5* was used for statistical analysis and plotting.

### Experimental details

For the validation of ML results, we have fabricated Ag/CuO/Pt/SiO_2_/Si device. Initially, the Si/SiO_2_ substrate was cleaned sequentially with bath-sonication in methanol, acetone, and distilled water, then dry with nitrogen gas. In the next step, the bottom Pt (500 nm) electrode was deposited on SiO_2_/Si substrate by RF sputtering technique. The CuO electrodeposition was carried out by a BioLogic's electrochemical workstation (VSP Potentiostat) on Pt/SiO_2_/Si substrate. In the typical electrodeposition method, a three-electrode system was used. In this case, the Pt helical coil, Ag/AgCl, and Pt/SiO_2_/Si substrate worked as a counter electrode, reference electrode, and working electrode, respectively. The electrolyte consisted of an aqueous solution of cupric nitrate and the acidic pH of the solution was adjusted by glacial acetic acid. The −0.1 V potential was applied for the deposition of CuO (~ 300 nm). The electrolyte temperature was maintained at 60 °C throughout the electrodeposition. For achieving a pure CuO state, the deposited film was annealed at 350 °C for 90 min. The top Ag (100 nm) electrode was deposited on CuO/Pt/SiO_2_/Si stack by RF sputtering technique. The electrical properties of the fabricated device were measured using a source measurement unit (Keithley 2602B) equipped with a manual probe station.

## Results and discussion

### Statistical analysis

**Figure 1:**
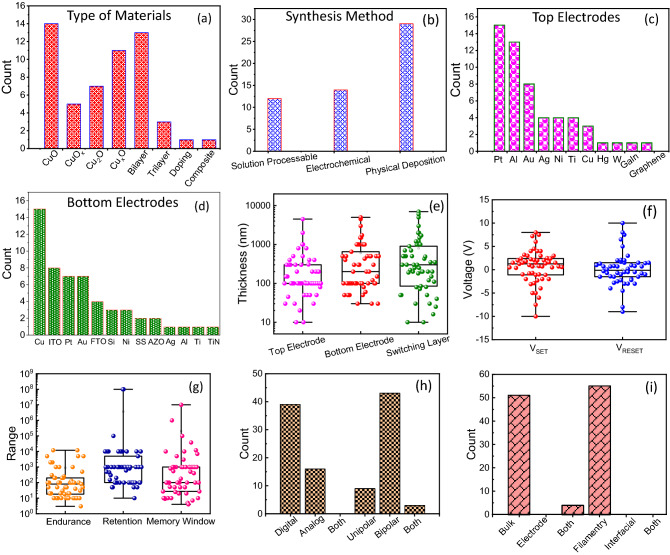
Statistical analysis of copper oxide-based RS devices. Bar plot of (**a**) type of materials used to fabricate RS layer and (**b**) synthesis methods used to deposit switching layer. Bar plot of types of (**c**) TE and (**d**) BE used to fabricate RS devices and their respective counts. (**e**) Thickness variation in TE, BE, and SL. Distribution of (**f**) switching voltages (V_SET_ and V_RESET_) and (**g**) endurance, retention, and memory window properties of RS devices. Bar plot of (**h**) type of resistive switching and (**i**) possible conduction and resistive switching mechanisms of RS devices.

In the present study, we examine the role of different process parameters on the copper oxide (CuO, CuO_x_, Cu_2_O, Cu_x_O), copper oxide-based bilayer, trilayer, and composite-based RS devices with the help of different ML techniques. The dataset required for ML was manually created by collecting data points from literature (2008–2022). The various synthesis parameters, structural parameters, and RS properties of devices used for ML analysis and predictions are summarized in Table S1. Statistical analysis of these parameters (continuous and categorical variables) is shown in Fig. 1.

The statistical analysis suggested that copper oxide was used as a switching layer (SL) in various manners. For instance, few reports used only copper oxide (CuO, CuO_x_, Cu_2_O, Cu_x_O) as a switching layer. In a few reports, copper oxide was used in the bilayer and trilayer device structures as an SL, while some devices were made up of copper oxide-based composites (Fig. 1a). Generally, copper oxide has two stable phases such as cupric oxide (CuO) and cuprous (Cu_2_O) oxide. In addition to these phases, few reports used copper oxide in the form of CuO_x_ and Cu_x_O as SL in RS devices. The results suggested that the CuO and Cu_x_O as SL were often used for fabricating RS devices (Fig. 1a) and they exhibit superior RS properties. In the case of deposition techniques, sputtering was the most preferred physical deposition technique. On the other hand, electrochemical deposition techniques were also used for the fabrication of SL, and very few reports were available on solution-processable copper oxide-based SLs (Fig. 1b). It is a well-known fact that the TE influences the RS properties. In particular, the RS properties of the electrochemical metallization memory and valance change memory devices strongly depend on the choice of TE^17,18^. Considering this, many electrochemically inert (Pt, Au, Hg, W, graphene), active (Ag, Cu), and oxidizable (Al, Ni, Ti) TEs were used by various research groups for the realization of RS devices. Figure 1c shows the previously used TEs for copper oxide-based RS devices. It is observed that Pt is frequently used as a TE^19^. The Al and Au were also used as TEs in a few reports. Likewise, the BE equally contributed to altering the RS characteristics. It is observed that the Cu metal was frequently preferred as a BE as compared to other metals for the fabrication of copper oxide-based RS devices (Fig. 1d). In the case of the electrochemical deposition (anodization) and thermal oxidation process, the Cu substrate is generally required for the growth of copper oxide and therefore, it acts as a BE in the desired device structure. Apart from Cu metal, the ITO, Pt, Au, FTO, and Si were often used as BE materials. Very few reports are available on the Ag, Al, Ti, and TiN electrode materials, as shown in Fig. 1d. The thickness of TE, BE, and SL is important factors that directly affect the RS properties. Figure 1e displays the thickness distribution of TE, BE, and SL. It is observed that the thickness of TE, BE, and SL are widely distributed from a few nanometers to a few micrometers. Therefore, this poses a challenge in optimizing the non-volatile memory properties. In the next ML section, we show how the thickness of these layers plays an important role in the RS process.

As per technology demand, the RS device should operate with minimum power consumption. Given this, the switching voltages (V_SET_ and V_RESET_) of the devices should be as minimum as possible. Figure 1f shows the V_SET_ and V_RESET_ distribution in copper oxide-based RS devices. The distribution of SET potential lies between + 8 and − 10 V and RESET potential lies between −9 and + 10 V. Generally, the conventional RS devices show positive bias SET and negative bias RESET properties. However, some devices switch oppositely due to their device structure, charge transport mechanism, and RS mechanism^20^. In the literature, the negative differential resistance (NDR) effect was also demonstrated for copper oxide-based RS devices^21^. Considering the technology demand, the research should be directed towards lowering the switching voltages. The non-volatile memory performance of any device is commonly benchmarked by endurance, retention, and memory window performance parameters. Figure 1g showcases the distribution of endurance, retention, and memory window of RS devices. To compete with industry-standard flash memory devices, the endurance of copper oxide-based RS devices should be ≥ 10^9^ to 10^10^^1^. Unfortunately, no report on copper oxide-based RS devices with endurance greater than 1.2 × 10^4^. In the retention case, retention time should be greater than 10 years and this criterion was fulfilled by only two devices from the literature^22,23^. In the case of RS devices, the memory window is at least in the order of 10^3^ or higher for better reading the resistive states^24^. From Fig. 1g, it is clear that a copper oxide-based RS device has the potential to show a memory window ≥ 10^3^. Interestingly, some devices show memory windows in the range of 10^5^ to 10^7^ (Fig. 1g).

In the case of switching type, most of the copper oxide-based RS devices either show digital type or analog type switching. In the case of digital-type switching, the device's current can abruptly increase or decrease under the influence of an external electric field. Therefore, such kind of devices is useful for non-volatile memory applications^25^. On the other hand, analog-type switching devices show gradual increase or decrease in the current and such property can be employed to mimic various synaptic learning properties^26^. The co-existence of both kinds of switching is not observed in these devices (Fig. 1h). Digital-type switching is frequently reported for copper oxide-based RS devices as compared to analog-type switching. Interestingly, unipolar, bipolar, and co-existence of both kinds of switching are observed in these devices. In particular, bipolar RS is more frequently reported than unipolar and a combination of both kinds of switching (Fig. 1h). In the case of the conduction mechanism, the bulk limited conduction mechanisms such as of Poole–Frenkel emission, hopping conduction, space-charge-limited conduction, etc. were reported in most of the devices. The electrode limited conduction is rarely reported for such devices (Fig. 1i). However, few reports demonstrated both kinds of switching mechanisms (bulk and electrode limited) in copper oxide-based RS devices (Fig. 1i). The statistical results suggested that most of the devices are based on the filamentary type RS mechanism. The interfacial and co-existence of both kinds of switching mechanisms were not reported by researchers (Fig. 1i).

### Design guidelines formulated using CART and DT-based ML algorithms

In this section, we formulate the guidelines for fabricating high-performance copper oxide-based RS devices based on the available dataset by CART and DT-based ML algorithms. The present dataset consists of both numerical and categorical features. Therefore, only one kind of ML algorithm is not suitable to get good fabrication guidelines. In this scenario, the CART is suitable for continuous (numerical) data^27^, whereas, the DT works very well on discrete data^28^. Considering these facts, we choose CART for continuous data (V_SET_, V_RESET_, EC, RT, and MW), whereas, DT was employed to handle categorical data (TSAD, TSUB, MRS, CM, and RSM). Initially, we selected the device performance parameters such as V_SET_, V_RESET,_ endurance, retention, and memory window to get an individual CART predictive model. For the CART algorithm, we provide the following input parameters: type of material (TYM), synthesis method (SM), TE, the thickness of TE (TTE), BE, the thickness of BE (TBE), and thickness of SL (TSL). Based on these input parameters, an individual CART predictive model was built for each performance parameter.

**Figure 2:**
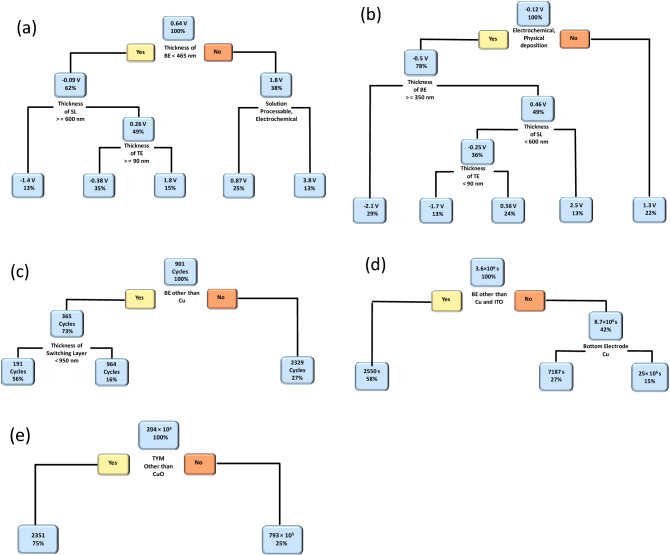
CART algorithm-based ML predictive model for copper oxide-based devices. The performance parameters such as (**a**) V_SET_ (V), (**b**) V_RESET_ (V), (**c**) endurance (cycles), (**d**) retention (s), and (**e**) memory window (unitless) are modeled using the CART-based ML algorithm.

Figure 2 shows the CART-based ML models of copper oxide-based RS devices. In the CART predictive model, different clusters are formed based on average values from the dataset for specific performance parameters called nodes. In the CART model, the first node is called the root node and other nodes are defined as leaf nodes. At each node, one can find the decision rule, which gives different fabrication parameters. Every decision node consists of a percentage value, which indicates the amount of data obeying that particular decision rule and the upper value represents the average value of that particular cluster. Initially, the CART algorithm was applied to V_SET_ voltages and the obtained model is shown in Fig. 2a. The model suggested that the TBE is the leading parameter, which directly impacts V_SET_ voltage_._ In particular, positive V_SET_ voltage can be achieved by maintaining the thickness of BE greater than 465 nm. While negative V_SET_ voltage can be achieved by maintaining the thickness of BE less than 465 nm. The literature suggested that conventional (positive V_SET_) and opposite (negative V_SET_) switching processes can be obtained in the RS devices, owing to the dominance of either oxygen vacancies^29^ or metallic Cu filaments^30^ during the formation and breaking of conductive filament(s), respectively. However, according to current ML predictions, TBE plays an important role in both conventional and opposite switching processes. Surprisingly, no experimental studies on TBE-dependent conventional and opposite switching processes for copper oxide-based RS devices have been reported, and this remains an open topic for interested researchers. The present model suggested that the electrochemical and solution-processable synthesis techniques are favorable for getting lower positive V_SET_ voltage than physical deposition techniques. Also, the model suggested that minimum negative V_SET_ voltage is attained by maintaining the thickness of SL < 600 nm and the thickness of TE less than 90 nm. Figure 2b depicted CART based V_RESET_ voltage prediction model of copper oxide-based RS devices. According to the proposed model, electrochemically and physical deposition-based copper oxide-based RS devices show a lower V_RESET_ voltage. On the other hand, the solution-processable RS devices show positive V_RESET_ voltage. In electrochemically and physically deposited devices, if we maintained the thickness of BE (< 350 nm), SL (< 600 nm), and TE (> 90 nm) of the RS device then it is possible to attain lower V_RESET_ voltage. It is interesting to note that both CART models (V_SET_ and V_RESET_) show evidence of conventional and unconventional switching voltage patterns i.e. positive V_SET–_negative V_RESET_ and negative V_SET–_positive V_RESET_. Such kind of physics-oriented model is very important to mimic the basic physical mechanism of electronic devices^31^.

Figure 2c shows the CART-based endurance prediction model of copper oxide-based RS devices. The model suggested the selection of the BE is a dominant parameter to get higher endurance values. If a device has Cu as a BE then the device will show maximum endurance (2329 cycles). The RS device with other BEs such as ITO, Pt, Au, FTO, Si, Ni, SS, AZO, Ag, Al, Ti, and TiN will result in lower endurance performance (365 cycles). The model also suggested that the thickness of SL plays an important role in endurance performance. If the thickness of SL is < 950 nm then the device shows an endurance of up to 191 cycles otherwise 964 cycles endurance can be achieved by the copper oxide-based RS devices.

Figure 2d shows the CART model of the memory retention property of copper oxide-based RS devices. Memory retention property is strongly dependent on BE rather than other input parameters. The maximum memory retention (25 × 10^6^ s) can be achieved by selecting Cu and ITO as the bottom electrodes. For the remaining BEs, the device can show a 2550 s memory retention property. Figure 2e depicted the CART model of the memory window of copper oxide-based RS devices. It is found that the memory window of the copper oxide-based RS devices is strongly dominated by the type of switching material. The model suggested that the phase pure CuO-based switching layer can provide a maximum memory window of 793 × 10^3^, as compared to other copper oxide materials (CuO_x_, Cu_2_O, Cu_x_O, bilayer, and trilayer).

**Figure 3:**
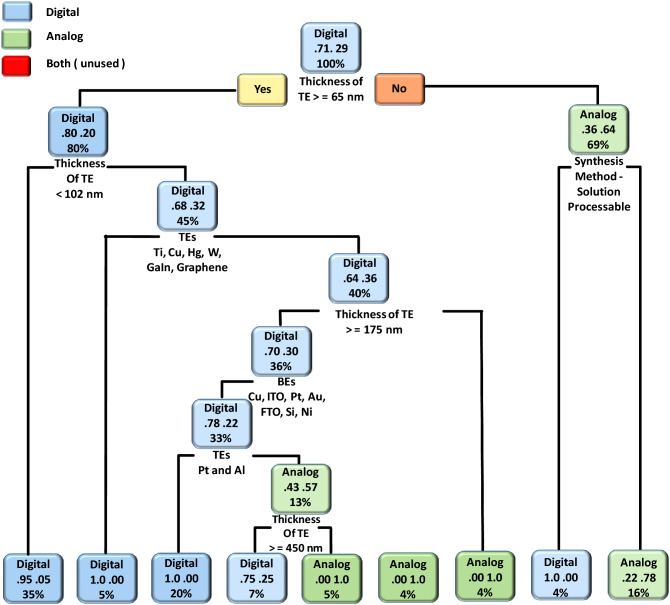
The DT model of the type of switching (analog, digital, or both). The present model suggests an impact of device fabrication parameters on the type of switching (analog, digital, or both) of copper oxide-based RS devices.

**Figure 4:**
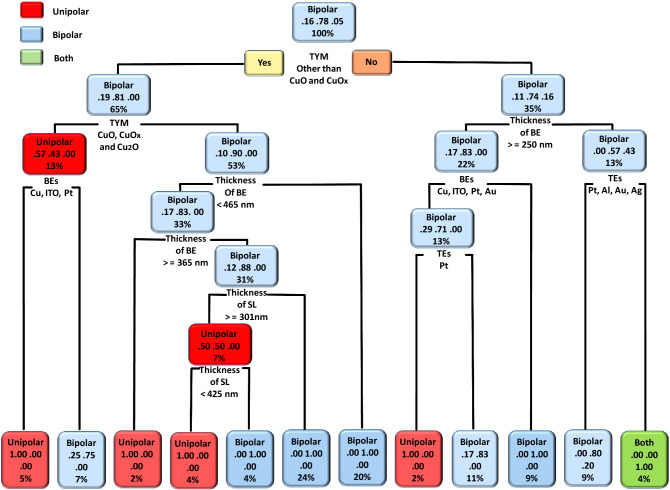
The DT model of the type of switching (unipolar, bipolar, or both). The present model suggests an impact of device fabrication parameters on the type of switching (unipolar, bipolar, or both) of copper oxide-based RS devices.

**Figure 5:**
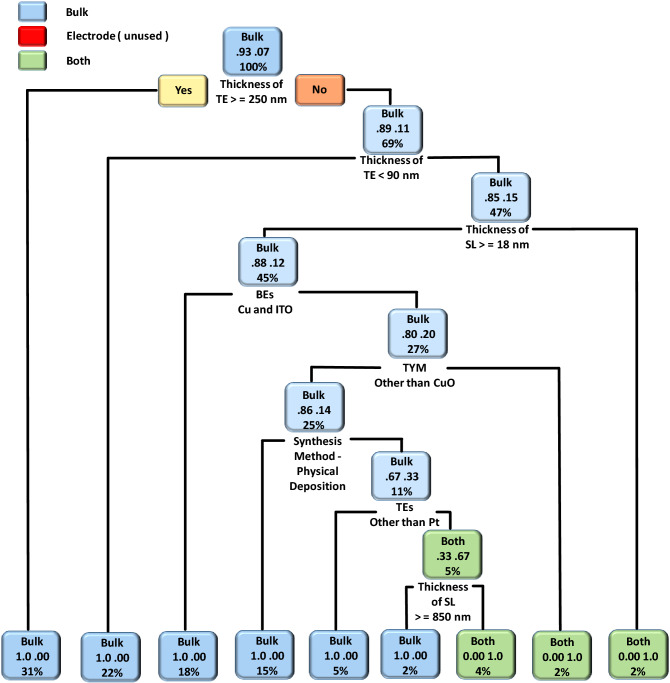
The DT model of the type of conduction mechanism (bulk limited, electrode limited, or both). The present model suggests an impact of device fabrication parameters on the type conduction mechanism of copper oxide-based RS devices.

To understand the effect of fabrication parameters on categorical features such as TSAD, TSUB, MRS, CM, and RSM, we used the DT ML algorithm. The obtained DT model results of copper oxide-based RS devices related to categorical features such as TSAD, TSUB, and CM are shown in Figs. 3, 4 and 5, respectively. In the case of MRS, most of the reported devices had shown two resistive states i.e. low resistance state (LRS) and high resistance state (HRS) during the non-volatile memory measurements. The intermediate resistance states are rarely reported for oxide-based RS devices. Moreover, the RS mechanism of most of the devices is based on filamentary effect and interfacial or co-existence of both mechanisms is rarely reported. Considering such predictable and unitary results, we have not provided DT models of the memory states and RS mechanism. In the DT model, the decision rules are placed at the bottom of each decision node (root or leaf node), the upper word represents the type of categorical feature, the middle number represents the probability of classes, and the percentage value represents the percentage of data that follows that particular decision rule.

Figure 3 represents the DT model of the TSAD. Among different device parameters, the thickness of TE plays an important role in the type of switching. For instance, if the thickness of TE is ≥ 65 nm then digital RS can be observed otherwise analog RS can be observed (if the thickness of TE is < 65 nm). In another node, the DT model suggested that the if the thickness of TE is < 102 nm then there is a 95% probability that the device shows digital switching. If it is > 102 nm then the probability of getting digital switching can be lowered to 68% and one can get analog switching with a probability of 32%. In the case of analog switching, synthesis methods play an important role. If the switching layer is fabricated using solution-processable techniques then the device can show digital type RS with 100% probability, however, only 4% of data follow such a trend. Therefore, it is always advised to use subject matter expertise while fabricating devices with ML-guided results^32^. If a device is fabricated using electrochemical and physical deposition techniques then there is a 78% probability to get analog switching. The present model further suggested that the choice of TEs, the thickness of TEs, and the choice of BEs influence the TSAD of copper oxide-based RS devices. The decision rules related to the co-existence of analog and digital type switching are not observed from the present DT model. This is due to the fact that such kind of data is not present in the prepared dataset.

The DT model results of the TSUB are shown in Fig. 4. The present model suggested that the bipolar RS is the most favorable switching type in the case of copper oxide-based RS devices. The following device parameters are important in the case of bipolar RS: type of material, the thickness of BEs, the thickness of SL, and the type of TEs and BEs. The unipolar and co-existence of both kinds of RS can be observed in copper oxide-based RS devices. However, such kind of switching can be complex to get and only 5% of data follow such kind of decision rules. In the most probable case (i.e. 5%), the unipolar switching can be observed by utilizing the following decision rule: type of material other than CuO and CuO_x_, type of material: Cu_2_O, and BEs: Cu, ITO, and Pt. The present model also suggested that the co-existence of unipolar and bipolar switching can be possible in copper oxide-based RS devices. Such kind of co-existence can be possible if the device is fabricated using the following decision rules: CuO and CuO_x_ as switching material, the thickness of BE < 250 nm, and TEs other than Pt, Al, Au, and Ag.

In the case of RS devices, the conduction mechanism is divided into three types viz. bulk limited, electrode limited, and co-existence of both. The DT model of the type conduction mechanism of copper oxide-based RS devices is shown in Fig. 5. The results asserted that the bulk-type conduction mechanisms (Poole–Frenkel emission, hopping conduction, space-charge-limited conduction, etc.) are dominated in the copper oxide-based RS devices. In some cases, the co-existence of both kinds of conduction mechanisms can be achieved by properly engineering the devices. For instance, the co-existence of both kinds of conduction mechanisms can be achieved by engineering the devices with the following parameters: the thickness of TE < 250 nm, the thickness of TE > 90 nm, and the thickness of the switching layer < 18 nm. The present DT model suggested that the bulk limited conduction mechanisms depend on the thickness and type of TEs, the thickness of SL, the type of BEs, the type of switching materials, and the synthesis method. Overall the CART and DT-based ML algorithms provide significant fabrication guidelines for the realization of optimized and high-performance copper oxide-based RS devices. These guidelines can be used by other researchers while optimizing copper oxide-based RS devices for non-volatile memory applications.

### ML predictions and feature selection

This section is associated with the prediction of different output features based on the device fabrication parameters. For the prediction of continuous output features, we have used ANN, LM, and RF algorithms. For the prediction of categorical output features, we employed the DT algorithm. A confusion matrix along with statistical measures was calculated by the DT algorithm. In the case of ML prediction of output features of devices (V_SET_, V_RESET_, EC, RT, and MW), the fabrication parameters of devices (TYM, SM, TE, TTE, BE, TBE, and TSL) were supplied to each ML algorithm and a corresponding ML model was built.

ANN is a special type of algorithm in ML that is inspired by a biological neural network and it is useful for the prediction of highly nonlinear data^33,34^. While LM is a regression-based algorithm and follows the linear approach for the complex data set^35^. The RF algorithm is an ensemble-based learning method and used multiple classifiers to solve complex problems^36^. For the predictive modeling, the random sampling method was used. In this method, the data were divided into a training dataset (70%) and a testing dataset (30%), and a cross-validation technique was used for model-building purposes. In the case of ANN, LM, and RF model predictions, the training data was used for the construction of the model, while testing data was used for evaluating the performance of these models.

**Figure 6:**
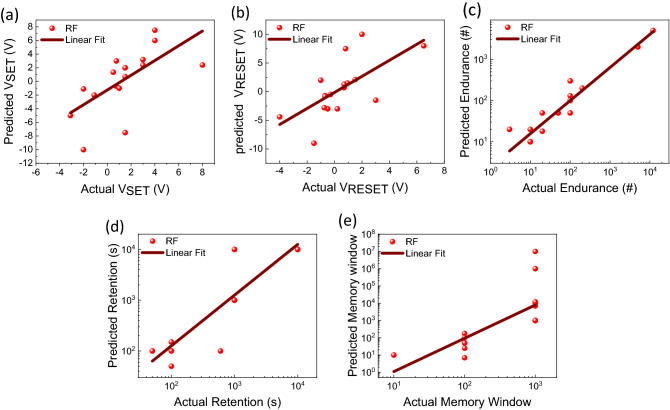
RF-based predictions of output performance features of the copper oxide-based RS devices. Predictions of (**a**) V_SET_, (**b**) V_RESET_, (**c**) endurance, (**d**) retention, and (**e**) memory window. The data are fitted by a linear fitting method.

Figure 6 shows the RF-based predictive model results related to output performance features of copper oxide-based RS devices. The ANN and LM-based predictive models of copper oxide-based RS devices are shown in Fig. S1. Statistical measures such as Pearson’s r and Adj. R^2^ values obtained from linear fitting decide the quality of predictions. The statistical measures of linear fitting of these models are summarized in Table S2. In the present case, Pearson’s r and Adj. R^2^ values of ANN and LM models are found to be very low, hence these models failed to predict the performance parameters of RS devices. However, Pearson’s r and Adj. R^2^ values of RF models are found to be high as compared to ANN and LM. The scattered plots of predicted and actual values of V_SET_, V_RESET_, EC, RT, and MW are also reasonably good, as shown in Fig. 6a–e. These results suggested that the RF algorithm can predict the output performance features of copper oxide-based RS devices based on the input features (fabrication parameters of devices). It should be noted that the limited amount of data points used in the present work can hammer the training and validation of the ML algorithms. Therefore, the robustness of present ML techniques can be improved by collecting a lot of data and incorporating them into future studies.

The categorical output features (TSUB, TSAD, and CM) are predicted by employing the DT algorithm. The confusion matrix with accuracy and misclassification of the DT algorithm for each output feature is shown in Figure S2. The confusion matrix represents the predicted and actual values of TSUB, TSAD, and CM. If all numerical values appear on the diagonal of the confusion matrix, then the DT model can correctly classify output features. In the case of TSAD, all numerical values are present at diagonal, suggesting the DT model correctly predicts the analog and digital type switching with 100% accuracy (Fig. S2a). In the case of TSUB and CM, one data point was wrongly classified/misclassified, as shown in Figure S2b,c, respectively. In the confusion matrix, the numerical values highlighted in red indicate a misprediction by the DT algorithm. For both the cases (TSUB and CM), the accuracy and misclassification are found to be 0.9411 (94.11%) and 0.0588 (5.88%), respectively. In the nutshell, the DT algorithm is quite good for the prediction of categorical output features of copper oxide-based RS devices.

**Figure 7:**
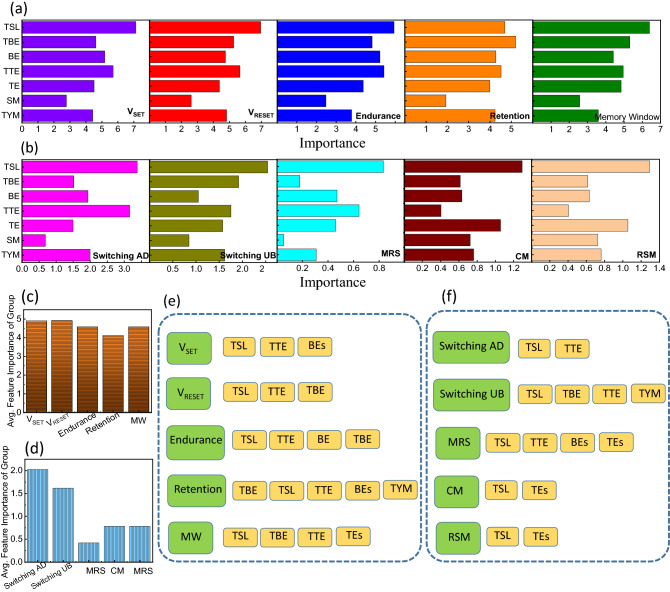
Feature importance score of the continuous and categorical output features. (**a**) Feature importance of continuous features (V_SET_, V_RESET_, EC, RT, and MW) and (**b**) categorical features (TSUB, TSAD, MRS, CM, and RSM). Average feature importance of (**c**) continuous features and (**d**) categorical features and (**e,f**) corresponding significant device fabrication parameters.

It is worthy to examine how input parameters impact the ML models of output features, therefore, we examine the feature importance ranking of each continuous and categorical input parameter/feature. For this, the GB algorithm was used. Figure 7a shows the feature importance score of V_SET_, V_RESET_, EC, RT, and MW (continuous features). Figure 7b shows the feature importance score of TSUB, TSAD, MRS, CM, and RMS (categorical features). From Fig. 7a it is observed that TSL, TTE, and TBE show comparatively high importance scores which directly impact V_SET_, V_RESET_, EC, RT, and MW. Among different input features, the synthesis method has the least importance score or least impact on the performance of V_SET_, V_RESET_, EC, RT, and MW (Fig. 7a). The categorical features (TSUB, TSAD, MRS, CM, and RSM) are largely influenced by the TTE and TSL. The synthesis method has the least impact on the TSUB, TSAD, and MRS. However, it relatively influences the CM and RMS. For finding crucial features that can impact device performance, we plot the graph of the average feature importance of the group for V_SET_, V_RESET_, EC, RT, and MW (Fig. 7c) and TSUB, TSAD, MRS, CM, and RSM (Fig. 7d). The significant input features are decided in a such way that the feature importance score of the input feature should be more than or equal to the average feature importance of the group.

Figure 7e,f show the significant continuous and categorical input features of copper oxide-based RS devices. In the case of switching voltages, the thickness of SL, TE, and BE are the major input features that can affect the device's performance. The V_SET_ is also influenced by BE materials. Similarly, endurance depends on the TSL, TTE, TBE, and BE materials. The retention property of copper oxide-based RS devices depends on the TSL, TTE, and TBE along with BE materials and the type of switching materials. It can be observed that only the memory window is affected by TE materials along with the TSL, TTE, and TBE. In the case of categorical features, the TSL and TTE influence the TSAD and TSUB. The TSUB also depends on the TBE and the type of switching materials. The MRS (Multiple Resistive States) can be influenced by the TSL, TTE, and the type of TE and BE. Both the CM and RSM are significantly affected by the TSL and the type of TE. In conclusion, we can say that each continuous and categorical input feature of copper oxide-based RS devices is highly dependent on the TSL and TTE.

### Experimental validation

For the validation of the present ML approach, we have fabricated a CuO-based RS device according to DT and CART-based decision rules, and the corresponding experimental conditions are provided in Table S3. The detailed device fabrication process is summarized in the experimental section. According to CART results shown in Fig. 2, minimum V_SET_ voltage (0.87 V) can be obtained by maintaining BE thickness > 465 nm and depositing the copper oxide layer by either solution processable or electrochemical synthesis techniques. The minimum V_RESET_ voltage (−0.5 V) can be obtained by depositing the copper oxide layer with either electrochemical or physical deposition techniques. In the case of endurance and retention, the CARTs suggested that the BE plays an important role. However, these CARTs cannot provide a unique set of rules. In the case of endurance, higher endurance (2329 cycles) can be obtained by using Cu as a BE. On the other hand, higher retention (25 × 10^6^ s) can be obtained by taking BE other than Cu. As retention property is more important than endurance, BE can be other than Cu metal. Last but not the least, a higher memory window (793 × 10^3^) can be observed by depositing the CuO switching layer. Therefore, we choose the following experimental conditions, BE: Pt, fabrication method: electrochemical, and Type of material: CuO. The remaining device parameters were chosen from the DTs, as shown in Figs. 3, 4 and 5. If the thickness of TEs is greater than 65 nm then digital-type switching can be observed. If the thickness of BE is greater than 465 nm and the thickness of SL is less than 301 nm then the bipolar RS can be observed. If TE is made up of other than Pt electrode then bulk limited conduction can be observed in the device.

**Figure 8:**
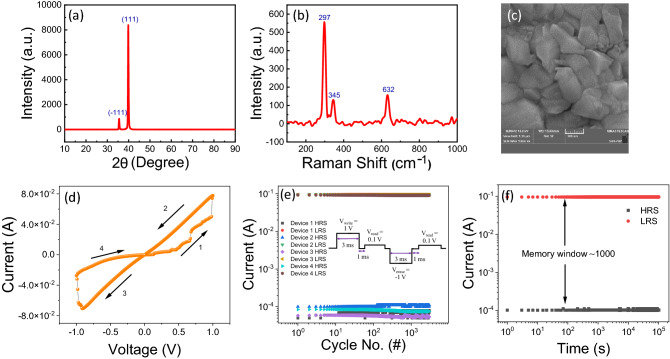
(**a**) XRD pattern, (**b**) Raman spectrum, and (**c**) FESEM image of fabricated CuO thin film. (**d**) I–V, (**e**) endurance, and (**f**) retention properties of CuO RS device.

The phase formation of the deposited switching layer was investigated by an X-Ray diffractometer (XRD, AXS D8 Advances, Bruker). Figure 8a depicted the XRD pattern of deposited CuO thin film. The diffracted peaks were observed at 35.50° and 39.50° corresponding to the (−111) and (111) planes. The result suggested that monoclinic CuO (JCPDS no. 89-5899) was deposited on Pt/SiO_2_/Si substrate^37,38^. Further confirmation of the CuO switching layer was carried out by the Raman spectroscopy technique (Renishaw INVIA0120-02). Figure 8b shows the Raman spectrum of deposited CuO thin film. The peak observed at 297, 345, and 632 cm^−1^ are due to the vibration motion of the Cu–O bond and are associated with A_g_ and B_g_ modes, respectively^39^. The morphological and compositional analysis of fabricated CuO thin film was analyzed by field emission scanning electron microscopy (FESEM, MIRA3 LMH, TESCAN) attached with energy dispersive spectroscopy (EDS) techniques. The microcrystal morphology was observed for electrodeposited CuO thin film, as shown in Fig. 8c. EDS spectra confirm the presence of Cu and O within fabricated thin films (Fig. S3). The appearance of Si and Pt is due to the use of the Pt/SiO_2_/Si substrate for the device fabrication.

Figure 8d,e,f show the I–V characteristics, endurance, and retention properties of the fabricated CuO RS device, respectively. The device shows reliable resistive switching properties as per the CART and DT results. The device shows SET/RESET processes within ± 1 V. The fabricated device shows good cyclic endurance (3000 cycles) and data retention (10^5^ s) properties. The pulse voltage stresses (PVS) based endurance measurement protocol is shown in the inset of Fig. 8e^40^. To understand the reliability of the proposed approach, we have measured the endurance property of 04 devices. All devices show similar kinds of endurance behavior, suggesting the good reliability of the CuO RS devices. The memory window (I_ON_/I_OFF_ ratio) of the fabricated device is ~ 1000.

It is interesting to note that not all experimental properties match the outcomes predicted by ML. The experimental results, however, are very close to those anticipated by ML. Such a deviation may occur because the DT delivers outcomes in terms of probability whereas the CART provides the average outcome value. Therefore, a small difference between the actual experimental and the ML projected results can be anticipated.

## Conclusion

In the present study, we collected data from previously reported peer-reviewed research articles for the analysis and performance prediction of copper oxide-based RS devices. The CART and DT algorithms provide several device fabrication guidelines for continuous and categorical performance features. Interestingly, the CART and DT unravel some of the unconventional switching voltage patterns of the copper oxide-based RS devices. Such kind of physics-oriented ML models is very important to mimic the basic physical mechanism of electronic devices. In the case of predictions of output features, the RF algorithm is most suitable for copper oxide-based RS devices as compared to LM and ANN. Moreover, the DT-based confusion matrix is quite suitable for the prediction of categorical output features RS devices with high accuracy and lower misclassification. The feature importance calculated by the GB algorithm reveals that all continuous and categorical performance features are highly dependent on the TSL and TTE. Finally, the ML-assisted fabricated CuO RS device shows good RS performance and these results are in good agreement with the ML predicted results. Overall ML prediction for copper oxide-based RS devices provides a strong knowledge base and optimized parameters for high-performance RS devices.

## Supplementary Information

Supplementary Information.

## Data Availability

The datasets generated during and/or analyzed during the current study are available from the corresponding author upon reasonable request.
