# Supplementary material for: Unraveling the importance of fabrication parameters of copper oxide-based resistive switching memory devices by machine learning techniques
Source: Sci Rep. 2023 Mar 25;13:4905. doi: 10.1038/s41598-023-32173-8 (PMC10039863; doi:10.1038/s41598-023-32173-8)
Supplement: Supplementary file 1 — Supplementary Information. [file 41598_2023_32173_MOESM1_ESM.docx]

**Supporting Information**

**Unraveling the Importance of Fabrication Parameters of Copper Oxide-based Resistive Switching Memory Devices by Machine Learning Techniques‡**

Suvarna M. Patil^1,†^, Somnath S. Kundale^2,†^, Santosh S. Sutar^3^, Pramod J. Patil^2^,

Aviraj M. Teli^4^, Sonali A. Beknalkar^4^, Rajanish K. Kamat^5,6^,

Jinho Bae^7^, Jae Cheol Shin^4,^*, Tukaram D. Dongale^2,^*

^1^ Institute of Management, Bharati Vidyapeeth Deemed to be University, Sangli 416 416, India

^2^ Computational Electronics and Nanoscience Research Laboratory, School of Nanoscience and Biotechnology, Shivaji University, Kolhapur 416004, India

^3^ Yashwantrao Chavan School of Rural Development, Shivaji University, Kolhapur 416004, India

^4^ Division of Electronics and Electrical Engineering, Dongguk University, Seoul 04620, South Korea

^5^ Department of Electronics, Shivaji University, Kolhapur 416004, India

^6^ Dr. Homi Bhabha State University, 15, Madam Cama Road, Mumbai-400032, India

^7^ Department of Ocean System Engineering, Jeju National University, 102 Jejudaehakro, Jeju 63243, South Korea

*Corresponding Authors

E-mail: tdd.snst@unishivaji.ac.in (Dr. Tukaram D. Dongale) and jcshin@dgu.ac.kr (Prof. Jae Cheol Shin)

†: These authors contributed equally to this work.

‡: This work is dedicated to Professor Pramod N. Vasambekar, in recognition of his outstanding research in the field of electronic materials and to commemorate his official superannuation from the Department of Electronics, Shivaji University, Kolhapur, India.

**Table S1:** Categorical features and variables used in the present study

| **Categorical features** | **Variables of the RS devices** |
| --- | --- |
| **Name of the RS Materials** | Copper Oxide |
| **Type of Materials** | CuO (14), CuO_x_ (5), Cu_2_O (7), Cu_x_O (11), Bilayer (13), Trilayer (3), Doping (1), Composite (1) |
| **Synthesis Methods** | Solution Processable (12), Electrochemical, (14) Physical Deposition (29) |
| **Top Electrodes** | Pt (15), Al (13), Au(8), Ag(4), Ni(4), Ti(4), Cu(3), Hg(1), W(1), GaIn (1), Graphene (1) |
| **Thickness of TEs (nm)** | Minimum 10 nm, Maximum 4500 nm |
| **Bottom Electrodes** | Cu (15), ITO (8), Pt (7), Au (7), FTO (4), Si (3), Ni (3), SS (2), AZO (2), Ag (1), Al (1), Ti (1), TiN (1) |
| **Thickness of BEs (nm)** | Minimum 30 nm, Maximum 500 0nm |
| **Thickness of Switching Layers (nm)** | Minimum 10 nm, Maximum 7000 nm |
| **Type of Switching** | Digital (39), Analog (16), Both (0) |
| **Type of Switching** | Unipolar (9), Bipolar (43), Both (3) |
| **SET Voltage (V)** | Minimum: -10, Maximum: 8 |
| **RESET Voltage (V)** | Minimum: -9, Maximum: 10 |
| **Endurance (#)** | Minimum: 3, Maximum: 1.2 × 10^4^ |
| **Retention (s)** | Minimum: 10, Maximum: 1 × 10^8^ |
| **Memory Window** | Minimum: 4, Maximum: 10^7^ |
| **Number of Memory States** | 2 and 3 states |
| **Application area** | Memory |
| **Conduction Mechanism** | Bulk limited (51), Electrode limited (0), Both (4) |
| **RS Mechanism** | Filamentary (55), Interfacial (0), Both (0) |
| **Year of publication** | 2008-2022 |

**Figure S1: ANN and LM-based predictions of output performance features of the copper oxide-based RS devices.** Predictions of (a) V_SET_, (b) V_RESET_, (c) endurance, (d) retention, and (e) memory window. The data are fitted by a linear fitting method.

**Table S2**: Linear fitting results of ANN, LM, and RF predictive models of copper oxide-based RS devices

| Copper oxide | | | | | | |
| --- | --- | --- | --- | --- | --- | --- |
|  | Pearson's r of ANN | Pearson's r of LM | Pearson's r of RF | Adj. R^2^ of ANN | Adj. R^2^ of LM | Adj. R^2^ of RF |
| V_SET_ | -0.0312 | -0.0549 | 0.6432 | -0.0822 | -0.0800 | 0.3746 |
| V_RESET_ | -0.4391 | -0.0703 | 0.6536 | 0.1255 | -0.0770 | 0.3890 |
| Endurance | -0.2842 | 0.3248 | 0.9558 | 0.0042 | 0.0061 | 0.9079 |
| Retention | 0.1541 | 0.3083 | 0.8865 | -0.0575 | 0.0196 | 0.7717 |
| Memory Window | -0.14303 | 0.17881 | 0.72552 | -0.0775 | -0.08903 | 0.4948 |

**Figure S2:** **DT algorithm-based predictions of categorical features of RS devices.** Confusion matrix, accuracy, and misclassification of (a) TSAD, (b) TSUB, and (c) CM output categorical features. The number shown in red color represents the wrong classification.

**Table S3**: DT and CART-based decision rules based experiment conditions

| Top electrode | Ag |
| --- | --- |
| Bottom electrode | Pt |
| Fabrication method | Electrochemical |
| Type of material | CuO |
| Thickness of the top electrode | 100 nm |
| Thickness of the bottom electrode | 500 nm |
| Thickness of switching layer | 100-300 nm |

**Figure S3.** EDS spectra of fabricated CuO thin film.
